# Enhancement of Antibiotic Activity by 1,8-Naphthyridine Derivatives against Multi-Resistant Bacterial Strains

**DOI:** 10.3390/molecules26237400

**Published:** 2021-12-06

**Authors:** José B. de Araújo-Neto, Maria M. C. da Silva, Cícera D. de M. Oliveira-Tintino, Iêda M. Begnini, Ricardo A. Rebelo, Luiz E. da Silva, Sandro L. Mireski, Michele C. Nasato, Maria I. L. Krautler, Jaime Ribeiro-Filho, Abolghasem Siyadatpanah, Polrat Wilairatana, Henrique D. M. Coutinho, Saulo R. Tintino

**Affiliations:** 1 Laboratory of Microbiology and Molecular Biology (LMBM), Regional University of Cariri—URCA, Crato 63105-000, CE, Brazil; jose.bezerra456@gmail.com (J.B.d.A.-N.); mariamilenecs@gmail.com (M.M.C.d.S.); datianemorais@hotmail.com (C.D.d.M.O.-T.); saulorelison@gmail.com (S.R.T.); 2 Department of Chemistry, Regional University of Blumenau—FURB, Itoupava Seca, Blumenau 89012-900, SC, Brazil; ieda@furb.br (I.M.B.); ricardorebelo@furb.br (R.A.R.); sandromireski@gmail.com (S.L.M.); michele_nasato_@hotmail.com (M.C.N.); maria.krautler@hotmail.com (M.I.L.K.); 3 Postgraduate Program in Sustainable Territorial Development—Coastal Sector, Federal University of Paraná, Curitiba 80060-000, PR, Brazil; luiz_everson@yahoo.de; 4 Gonçalo Moniz Institute, Oswaldo Cruz Foundation (IGM-FIOCRUZ/BA), Rua Waldemar Falcão, 121, Candeal, Salvador 40296-710, BA, Brazil; jaimeribeirofilho@gmail.com; 5 Ferdows School of Paramedical and Health, Birjand University of Medical Sciences, Birjand 9717853577, Iran; 6 Infectious Diseases Research Center, Birjand University of Medical Sciences, Birjand 9717853577, Iran; 7 Department of Clinical Tropical Medicine, Faculty of Tropical Medicine, Mahidol University, Bangkok 10400, Thailand

**Keywords:** naphthyridine, naphthyridinone, fluoroquinolones, bacterial resistance, synergism

## Abstract

The search for new antibacterial agents has become urgent due to the exponential growth of bacterial resistance to antibiotics. Nitrogen-containing heterocycles such as 1,8-naphthyridine derivatives have been shown to have excellent antimicrobial properties. Therefore, the purpose of this study was to evaluate the antibacterial and antibiotic-modulating activities of 1,8-naphthyridine derivatives against multi-resistant bacterial strains. The broth microdilution method was used to determine the minimum inhibitory concentration (MIC) of the following compounds: 7-acetamido-1,8-naphthyridin-4(1*H*)-one and 3-trifluoromethyl-*N*-(5-chloro-1,8-naphthyridin-2-yl)-benzenesulfonamide. The antibiotic-modulating activity was analyzed using subinhibitory concentrations (MIC/8) of these compounds in combination with norfloxacin, ofloxacin, and lomefloxacin. Multi-resistant strains of *Escherichia coli*, *Pseudomonas aeruginosa*, and *Staphylococcus aureus* were used in both tests. Although the compounds had no direct antibacterial activity (MIC ≥ 1.024 µg/mL), they could decrease the MIC of these fluoroquinolones, indicating synergism was obtained from the association of the compounds. These results suggest the existence of a structure–activity relationship in this group of compounds with regard to the modulation of antibiotic activity. Therefore, we conclude that 1,8-naphthyridine derivatives potentiate the activity of fluoroquinolone antibiotics against multi-resistant bacterial strains, and thereby interesting candidates for the development of drugs against bacterial infections caused by multidrug resistant strains.

## 1. Introduction

Bacterial diseases have been associated with high mortality rates at various times in history, and the discovery of substances with potential for preventing and healing bacterial infections has had a significant impact on science and public health [1]. In this context, antibiotics are crucial products for modern medicine, contributing to increased life expectancy and reduced child mortality. They also have fundamental applications in surgical processes and chemotherapy [2].

Multidrug resistance in bacteria has become a global epidemic [3]. *Pseudomonas aeruginosa* (*P. aeruginosa*), *Escherichia coli* (*E. coli*), and *Staphylococcus aureus* (*S. aureus*) are opportunistic pathogens that are among the most clinically and epidemiologically relevant multi-resistant bacteria [4]. Bacterial resistance mechanisms can be classified as intrinsic or acquired. While intrinsic mechanisms are related to structural or functional characteristics of the microorganism, acquired mechanisms result from mutations or horizontal gene transfer [5,6].

The management of infections caused by multi-resistant microorganisms is challenging because the emergence of new resistant organisms every day demands the development of new antibacterial compounds since current antibiotics are not enough [7]. The search for bioactive substances has identified a class of molecules with enormous therapeutic potential known as heterocyclic compounds [8]. This class of molecules represents about half of all known compounds including drugs, vitamins, and bioactive metabolites of plants and marine organisms [9].

Heterocyclic compounds are estimated to account for about 62% of active pharmaceutical ingredients. Among these substances, 91% are nitrogen-containing compounds. Nitrogen-containing heterocycles include both chemically synthesized and naturally occurring compounds such as 1,8-naphthyridine derivatives [10]. Studies have shown that these compounds have various biological activities such as antibacterial [11], antifungal [12], antiviral [13], anti-inflammatory [14], antitumor [15], and analgesic [16], and are therefore promising candidates for the development of new drugs.

Therefore, the present study aimed to evaluate the antibacterial and antibiotic-modulating activity of 1,8-naphthyridine derivatives against multi-resistant bacterial strains.

## 2. Results

### 2.1. Antibacterial Activity

An analysis of the antibacterial activity of 7-acetamido-1,8-naphthyridin-4(1*H*)-one (1,8-NA) and 3-trifluoromethyl-*N*-(5-chloro-1,8-naphthyridin- 2-yl)-benzenesulfonamide (3-TNB) against the multi-resistant strains *E. coli* 06, *S. aureus* 10, and *P. aeruginosa* 24 revealed that both compounds had minimum inhibitory concentrations (MICs) ≥ 1.024 µg/mL against all tested strains, indicating that they have no clinically relevant antibacterial activity.

### 2.2. Antibiotic-Modulating Activity

**Figure 1:**
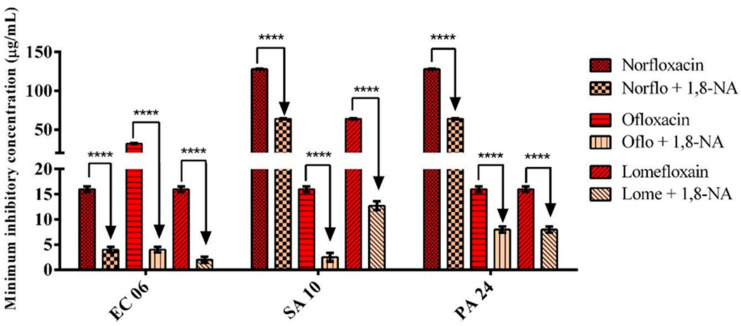
Antibiotic-modulating activity of 7-acetamido-1,8-naphthyridin-4(1*H*)-one (1,8-NA) in combination with norfloxacin, ofloxacin, and lomefloxacin against *E. coli* 06, *S. aureus* 10, and *P. aeruginosa* 24. **** Statistical significance: *p* < 0.0001.

Having demonstrated that 1,8-NA presented no clinically relevant antibacterial activity against any of the strains tested, we evaluated the modulatory action of this substance in combination with norfloxacin, ofloxacin, and lomefloxacin fluoroquinolones. As shown in Figure 1, in the presence of subinhibitory concentrations of 1,8-NA, all antibiotics showed significantly reduced MICs against multi-resistant strains *S. aureus* 10, *P. aeruginosa* 24, and *E. coli* 06, indicating that the combination of antibiotics with the 1,8-naphthyridine derivative showed synergism with regard to the antibacterial activity. The greatest reductions in antibiotic MICs occurred with ofloxacin (32 to 4 µg/mL) and lomefloxacin (16 to 2 µg/mL), both against *E. coli* 06.

**Figure 2:**
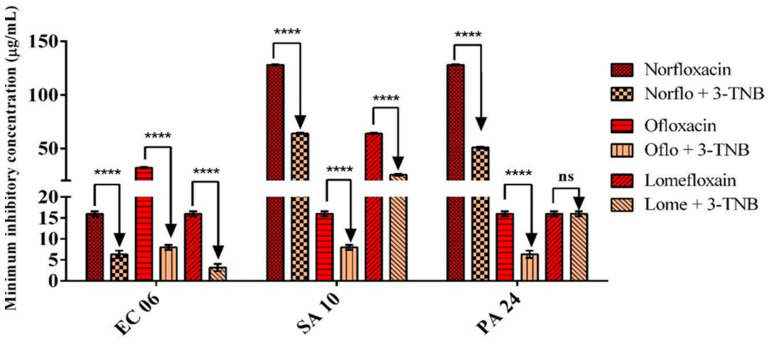
Antibiotic-modulating activity of 3-trifluoromethyl-*N*-(5-chloro-1,8-naphthyridin-2-yl)-benzenesulfonamide (3-TNB) in combination with norfloxacin, ofloxacin, and lomefloxacin against *E. coli* 06, *S. aureus* 10, and *P. aeruginosa* 24. **** Statistical significance: *p* < 0.0001; ns: not significant.

Similarly, the combination of 3-TNB with antibiotics under the same conditions described above caused a significant reduction in MIC of these drugs, except for the association with lomefloxacin against *P. aeruginosa* 24 (Figure 2). The greatest reduction occurred in the MIC of lomefloxacin (16 to 3.2 µg/mL) against *E. coli* 06. Taken together, our results indicate that the 1,8-naphthyridine derivatives evaluated by the present study are capable of modulating fluoroquinolone activity against both Gram-positive and Gram-negative multi-resistant bacteria.

## 3. Discussion

Due to a broad spectrum of biological actions previously mentioned, 1,8-naphthyridine derivatives have gained increasing importance in the fields of medicinal chemistry and drug development. Studies indicate that these compounds have enormous potential for the synthesis of new molecules, which has been associated with the structure–activity relationship in several biological models [11,12,13,14,15,16]. Furthermore, molecular modifications can be used to improve the physicochemical properties of these substances, generating compounds with optimized therapeutic effects [8,9,10].

The study carried out by Abu-Melha et al. [17] evaluated the antimicrobial potential of nitrogen-containing synthetic heterocycles and demonstrated that these compounds did not have a direct antibacterial action. According to the authors, using the agar diffusion method, no clinically relevant antibacterial activity was detected against *S. aureus* and *E. coli* given the fact that the MICs of the compounds were higher than the MIC of the gentamicin control (aminoglycoside). Studies with naphthyridine compounds showed similar results, with no significant antibacterial activity being obtained, which corroborates the data obtained in the present study [18,19]. Here, we demonstrated that the 1,8-naphthyridine derivatives 1,8-NA and 3-TNB presented MICs ≥1.024 µg/mL, and as such, are not promising antibacterial agents, as a higher concentration could lead to significant toxicity [20].

Insertion of fluoroquinolones as antibacterial agents in the last century has represented a breakthrough in antibiotic therapy, mainly due to their efficacy in combating infectious diseases [21]. Although these drugs were initially used only for the treatment of Gram-negative bacterial infections, the improvement in their properties allowed for the therapeutic use of these drugs to treat Gram-positive infections [22]. These antibiotics inhibit the activity of DNA gyrase (or topoisomerase II), an enzyme that is essential for bacterial survival. This enzyme acts by making the DNA molecule compact and biologically active. Thus, when this enzyme is inhibited, the DNA molecule free ends determine the uncontrolled synthesis of messenger RNA and proteins, causing bacterial death [23].

The data described by the present study demonstrate that fluoroquinolone association with 1,8-NA and 3-TNB significantly enhanced the antibacterial action of this class of antibiotics. This further suggests that 1,8-naphthyridine derivatives and antibiotics interact chemically, resulting in a synergistic antibacterial action. In fact, the radicals of these compounds can interact with various sites in the bacterial cellular environment. In addition, it is important to mention that 1,8-NA is employed in the synthesis of 1,8-naphthyridines such as 3-TNB [24], from which nalidixic acid was synthesized. This compound was a prototype for quinolones, which posteriorly originated fluoroquinolones [25].

A study showed that 1,5-naphthyridinone derivatives act by inhibiting bacterial topoisomerase, causing bacterial death [26]. Another study also reported on enzymatic inhibition as a potential antibacterial effect of naphthyridinones against strains of *E. coli* and *S. aureus* [27]. Eweas et al. [28] showed that 1,8-naphthyridine derivatives have the ability to bind topoisomerase II enzymes and can inhibit them as well as fluoroquinolones. These findings support the evidence of a synergistic interaction between 1,8-NA and 3-TNB with fluoroquinolones based on their similar antibacterial mechanisms of action.

In addition to the research cited, using in silico studies, Gençer et al. [29] demonstrated that 1,8-naphthyridine derivatives inhibit DNA gyrase (topoisomerase) such as fluoroquinolones, and through in vitro studies, they obtained a significant antibacterial effect against Gram-positive and Gram-negative strains. However, the authors worked with strains susceptible to antibiotics, which explains the difference in the results above-mentioned and gives us clues that the structural similarity between the 1,8-naphthyridine derivatives and the fluoroquinolones causes some resistance to the tested compounds.

Da Silva et al. [30], using the same bacterial strains and antibiotics of the present research, demonstrated that the Meldrum’s acid arylamino methylene derivative *N*-{6-[(2,2-dimethyl-4,6-dioxo-[1,3]-dioxane-5-ylidenomethyl)-amino]-pyridin-2-yl}-acetamide enhanced the effect of fluoroquinolones, attributing the synergistic effect to the similarity between the mechanism of action of its compound and the respective antibiotics. In addition to the fact that 1,8-naphthyridine derivatives and fluoroquinolones share similarities in chemical structure and mechanism of action on bacteria, these derivatives have been shown to inhibit bacterial resistance mechanisms [31]. Efflux pumps are transmembrane proteins that remove compounds toxic to bacteria from the intracellular medium and are one of the main mechanisms of resistance to fluoroquinolones [32]. 1,8-Naphthyridine derivatives have already been reported as inhibitors of NorA and MepA efflux pumps, which efflux norfloxacin and ciprofloxacin, respectively [33,34]. These results provide new insights into the antibiotic-modulating activity of 1,8-naphthyridine derivatives demonstrated in the present research.

Naphthyridines may occur in six different isomeric forms: 1,5-naphthyridine, 1,6-naphthyridine, 1,7-naphthyridine, 1,8-naphthyridine, 2,6-naphthyridine, and 2,7-naphthyridine [25]. Among these, due to scientifically proven biological activities, 1,8-naphthyridines are the most studied. It was indicated that these compounds have a wide variety of biological activities such as antiparasitic, antibacterial, anti-inflammatory, antiallergic, and antitumor [35]. According to Leonard et al. [36], these compounds stand out for their antimicrobial potential, which can be potentiated by the combination with sulfonamides [37].

Our research group has shown that the association between different substances does not always result in potentiated antibacterial activity. In fact, drug combinations can result in reduced drug bioactivity, either by mutual chelation [38], or by competition for the binding site in the microorganism [39]. In this case, an increased minimum inhibitory concentration was observed, indicating that the association has no clinical benefit. Some associations, however, are neutral (i.e., there is no synergism or antagonism) [40]. This phenomenon could explain the results obtained from the combination of lomefloxacin with naphthyridine 3-TNB against *P. aeruginosa*, which was found to be an exception to the significant antibiotic-modulating effects exerted by 1,8-naphthyridine derivatives in the present study.

## 4. Materials and Methods

### 4.1. Obtaining and Preparation of Nitrogen-Containing Heterocycles and Antibiotics

**Figure 3:**
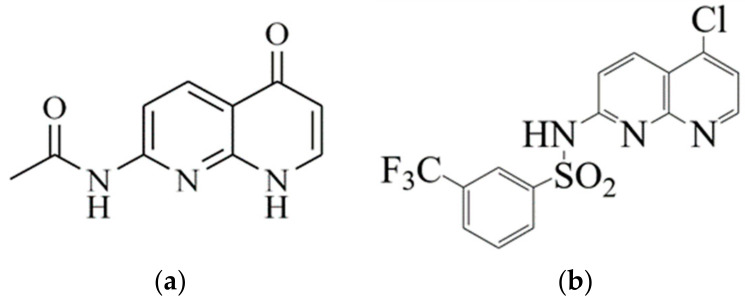
Chemical structures of the 1,8-naphthyridine derivatives: (**a**) 7-acetamido-1,8-naphthyridin-4(1H)-one (1,8-NA); (**b**) 3-trifluoromethyl-N-(5-chloro-1,8-naphthyridin-2-yl)-benzenesulfonamide (3-TNB).

The following compounds: 7-Acetamido-1,8-naphthyridin-4(1*H*)-one (1,8-NA) and 3-trifluoromethyl-*N*-(5-chloro-1,8-naphthyridin-2-yl)-benzenesulfonamide (3-TNB) (Figure 3), provided by Dr. Luiz Everson da Silva of the Federal University of Paraná, were obtained by chemical synthesis. The FTIR SPECTRUM, NMR ^1^H and ^13^C besides 19F of 7-Acetamido-1,8-naphthyridin-4(1*H*)-one (1,8-NA) and 3-trifluoromethyl-*N*-(5-chloro-1,8-naphthyridin-2-yl)-benzenesulfonamide (3-TNB) are supplied in the Supplementary Materials. The chemical synthesis occurred from the thermolysis of Meldrum’s acid adduct. The final sulfonamide derivatives were obtained by sulfonylation of 2-amino-5-chloro-1,8-naphthyridine with different commercial benzenesulfonyl chlorides [34].

**Figure 4:**
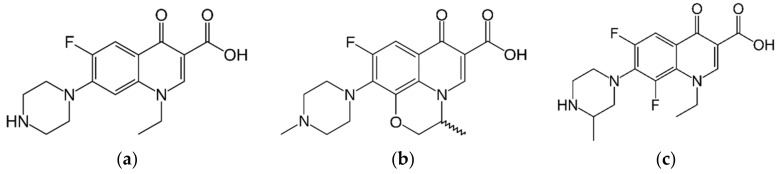
Chemical structures of the fluoroquinolones: (**a**) norfloxacin; (**b**) ofloxacin; (**c**) lomefloxacin.

The fluoroquinolones norfloxacin, ofloxacin, and lomefloxacin (Figure 4) were purchased from SIGMA Chemical (St. Louis, MI, USA), and were chosen for their strong relationship with the tested compounds, which are their precursors. A total of 10 mg of each substance was diluted in 1 mL of dimethyl sulfoxide (DMSO) to a concentration of 10 mg/mL, which was diluted in sterile distilled water to a 1.024 µg/mL [41].

### 4.2. Bacterial Strains and Preparation of Inocula

The following multi-resistant strains were used in the tests: *E. coli* 06, *S. aureus* 10, and *P. aeruginosa* 24. The origin and resistance profile of these strains were described by Bezerra et al. [42]. These bacteria were supplied by the Laboratory of Microbiology and Molecular Biology (LMBM) of the Regional University of Cariri (URCA), kept on blood agar (Laboratory Difco Ltd., Crato, Brazil) and cultured in heart infusion agar medium (HIA, Difco. Laboratorises Ltd.) at 37 °C for 24 h.

Inocula were prepared by taking a sample from the corresponding culture and adding it to tubes containing 5 mL of sterile saline (0.9% NaCl). The tubes containing the bacterial suspensions were shaken, and their turbidities were compared to the 0.5 McFarland scale, corresponding to 1.5 × 10^8^ colony forming units (CFU)/mL [43].

### 4.3. Minimum Inhibitory Concentration (MIC)

Following the methodology of Javadpour et al. [44], 100 µL of the inoculum was added to tubes containing 900 µL of the 10% BHI (Brain Heart Infusion Broth). Aliquots of 100 µL of each solution were transferred to a 96-well plate, followed by serial dilution (1:1) of the compounds to reach concentrations ranging from 512 to 8 µg/mL in the plates. The last well was added with the inoculum in the absence of treatment and was used as bacterial growth control. The assays were performed in triplicate, and plates were incubated in a bacterial greenhouse at 37 °C. After 24 h, 20 µL of resazurin (7-hydroxy-3H-phenoxazine-3-one-10-oxide) at 0.4 mg/mL was added to each well and, after 1 h at 25 °C, a color change from blue to pink indicated bacterial growth, by redox reaction, with the reaction of acid released by the bacteria with resazurin.

### 4.4. Evaluation of the Antibiotic-Modulating Activity

Briefly, 1.162 µL of 10% BHI, 150 µL of inoculum from each strain, and a volume of the compound corresponding to the subinhibitory concentration (MIC/8 = 128 µg/mL) were added to test tubes, and a subinhibitory concentration was used to assess whether the substances potentiated the effect of fluoroquinolones. Modulation controls were prepared using only 1.350 µL of 10% BHI medium and 150 µL of inoculum. The contents of each tube were distributed in 96-well plates (100 µL/well). Microdilution (1:1) was performed by adding 100 µL of each antibiotic whose concentrations in the plates ranged from 512 to 0.5 µg/mL [45]. The tests were performed in triplicate and the plates incubated at 37 °C for 24 h. Readings were taken 1 h after the addition of resazurin, as previously described.

### 4.5. Statistical Analysis

The means of the three repetitions were submitted to two-way analysis of variance (ANOVA) followed by Bonferroni’s post-hoc test using GraphPad Prism software, version 5.0. Results were considered significant when differences between groups (*p*) were <0.05.

## 5. Conclusions

From the results of the present research, it is possible to conclude that the 1,8-naphthyridine derivatives 1,8-NA and 3-TNB do not have intrinsic antibacterial activity against multi-resistant strains. Despite this, the compounds potentiate the effect of fluoroquinolones against *E. coli* 06, *S. aureus* 10, and *P. aeruginosa* 24, and can therefore be considered as adjuvants to antibacterial drugs. Existing evidence suggests that synergistic effects are related to similarity in chemical structure and mechanisms of action.

## Data Availability

All data and analyses are available in the manuscript.

## Supplementary Materials

The following are available online, 7-Acetamido-1,8-naphthyridin-4(1H)-one (1,8-NA) and 3-trifluoromethyl-N-(5-chloro-1,8-naphthyridin-2-yl)-benzenesulfonamide (3-TNB): FTIR SPECTRUM, NMR 1H and 13C besides 19F.

## Sample Availability

Samples of the compounds are available from the authors.
