# Supplementary material for: Enhancement of Antibiotic Activity by 1,8-Naphthyridine Derivatives against Multi-Resistant Bacterial Strains
Source: Molecules. 2021 Dec 6;26(23):7400. doi: 10.3390/molecules26237400 (PMC8659213; doi:10.3390/molecules26237400)
Supplement: Supplementary file 1 [file molecules-26-07400-s001.zip › molecules-1488473-supplementary.pdf]

Supplementary Data

7-Acetamido-1,8-naphthyridin-4 (1*H*)-one: Brown solid. Yield: 84%; mp: 298-299 °C. IR (KBr, cm^{-1}): 3331 (◎$_{N-H}$); 2953 (◎$_{C-H}$); 1674 (◎$_{C=O}$); 1611 (◎$_{C=O}$); 1516; 1306; 1196; 835; 598. ^{1}H NMR: ($CDCl_3$, 300 MHz, δ ppm): 2.14 (s, 3H, -CH_3); 6.02 (d, *J*=7,6 Hz, 1H, CH_{Ar}); 7.80 (d, *J*=7,6 Hz, 1H, CH_{Ar}); 8.03 (d, *J*=8,8 Hz, 1H, CH_{Ar}); 8.37 (d, *J*=8,4 Hz, 1H, CH_{Ar}); 10.69 (s, 1H, -NH); 11.64 (d, *J*=4,8 Hz, 1H, -NH). ^{13}C NMR: ($CDCl_3$, 75 MHz, δ ppm): 24.91 (-CH_3); 110.50; 111.09; 117.53; 137.68; 140.47; 150.20; 154.78; 170.76 (-C=O); 177.59 (-C=O).

3-Trifluoromethyl-*N*-(5-chloro-1,8-naphthyridin-2-yl)-benzenesulfonamide: Light yellow solid. Yield: 53%, mp: 222.2-224 °C. IR (ATR/FTIR, cm^{-1}): 3265 $_{(N-H)}$, 3066 $_{(=CH)}$, 1645 $_{(N-H)}$, 1600, 1534, 1468 and 1410 $_{(C=C)}$, 1323 $_{(as\ S=O)}$, 1122 $_{(s\ S=O)}$, 1100 $_{(Ar-Cl)}$, 826 $_{(=CH)}$. ^{1}H NMR (DMSO-*d6*, 300 MHz, δ ppm): 7.33 (d, *J*=9.72 Hz, 1 H, CH_{AR}); 7.50 (d, *J*=5.14 Hz, 1 H, CH_{AR}); 7.71-7.78 (m, 1 H, CH_{AR}); 7.88-7.93 (m, 1 H, CH_{AR}); 8.19-8.27 (m, 2H, CH_{AR}); 8.30 (d, *J*=9.72 Hz, 1 H, CH_{AR}); 8.59 (d, *J*=5.14 Hz, 1 H, CH_{AR}); 11.47 (s, 1 H, N-H). ^{13}C NMR (DMSO-*d6*, 75 MHz, δ ppm): 107.83; 112.65; 112.88; 113.26; 121.36; 121.97; 124.67; 125.58; 129.51; 129.94; 130.37; 130.81; 131.29; 133.65; 136.65; 137.50; 140.86; 144.10; 149.05; 154.98; 172.95. ^{19}F NMR (DMSO-*d6*, 282 MHz, δ ppm): -58.5 $_{(\nu\ Ar-CF3)}$. HR-MS (ESI, TOF): $C_{15}H_9N_3O_2SClF_3$, theoretical $[M]^+$ *m*/*z*=388.0130; experimental $[M]^+$ *m*/*z*=388.0129.
